# Cytokine release syndrome after chimeric antigen receptor T cell therapy in patients with diffuse large B-cell lymphoma: a systematic review

**DOI:** 10.1016/j.htct.2024.05.005

**Published:** 2024-07-23

**Authors:** Andressa Rodrigues dos Santos, Daniela Zanini, Daniel Andolfatto

**Affiliations:** Associação Hospitalar Lenoir Vargas Ferreira, Chapecó, SC, Brazil

**Keywords:** Chimeric antigen receptor, Cytokine release syndromes, Diffuse large B Cell lymphoma, Adoptive cell transfer, T-cell receptor genes

## Abstract

**Introduction:**

Chimeric antigen receptor T (CAR-T) cell therapy is an innovative technology that has shown promising results in clinical trials. Treatment is based on modifying the patient's own T cells to express artificial surface receptors to specifically recognize and attack the tumor cells.

**Objective:**

To synthesize available evidence on the incidence and management strategies of cytokine release syndrome in patients with diffuse large B-cell lymphoma who received CAR-T cell therapy.

**Methods:**

This is a systematic literature review. The search was conducted in the PubMed, Scopus, and Web of science databases. This review followed the Preferred Reporting Items for Systematic Reviews and Meta-analyses (PRISMA) guidelines. The systematic review protocol is registered in the International Prospective Register of Systematic Reviews (PROSPERO) database under number CRD42022359258.

**Results:**

Nineteen studies were included with a total of 1193 patients who received CAR-T cell therapy. Of these patients, 804 (67%) developed some degree of cytokine release syndrome. The frequencies of Grade 3 and 4 cytokine release syndrome were 10% and 3%, respectively. The regimen most used in the management of the syndrome included tocilizumab and/or glucocorticoids.

**Conclusion:**

The results obtained in this review demonstrate high rates of cytokine release syndrome in patients with diffuse large B-cell lymphoma treated with CAR-T cell therapy, however these events are manageable, supporting the conclusion that this therapy is safe in these patients.

## Introduction

For many decades, cancer therapy was based mainly on surgery, chemotherapy and radiotherapy. In recent years, therapeutic approaches based on stimulating the patient's immune response have established cancer immunotherapies as new treatment options.^1^ Immunotherapy with chimeric antigen receptor T (CAR-T) cells, is an innovative technology that has shown promising results in clinical trials in patients presenting with relapsed or refractory B-cell hematologic neoplasms.^2^^,^^3^

The mechanism of action of autologous CAR-T cells is based on modifying the patient's own T cells to express receptors for a target antigen. In this type of treatment, blood is collected through leukapheresis and T cells are genetically modified and reinfused into the patient.^4^ These modified T cells contain chimeric antigen receptors, which are artificial surface receptors that aim to specifically recognize and attack the patient's tumor.^5^

The first paper on the chimeric combination of receptors and antibodies was published in 1989 by the Weizmann Institute in Israel.^6^ Since then effort has been devoted to this area of research, leading to therapeutic success in 2012, when seven-year-old Emily Whitehead was cured of relapsed and refractory B-cell acute lymphoblastic leukemia by CAR-T cell therapy.^7^^,^^8^

The therapy has shown promise and despite advances, many clinical trials involving CAR-T continue to be carried out in order to ensure greater safety and efficacy.^9^ Common adverse effects related to the use of CAR-T cell therapy, such as cytokine release syndrome (CRS), may limit its use.

Thus, a synthesis of evidence is needed to evaluate the safety of these therapies.^10^ The aim of this study is to synthesize available evidence in the scientific medical literature on the occurrence and management strategies of CRS in patients with diffuse large B-cell lymphoma (DLBCL) who received CAR-T cell therapy.

## Methods

### Research strategy

This is a systematic literature review in which all data were extracted from the published literature, so no ethical review or patient consent was required. This review followed the Preferred Reporting Items for Systematic Reviews and Meta-analyses (PRISMA) guidelines. The systematic review protocol is registered in the International Prospective Register of Systematic Reviews (PROSPERO) database under number CRD42022359258.

The search was conducted in the PubMed, Scopus, and Web of science databases. Articles published in English up to September 2022 were eligible. The search terms and Boolean operands used were: ‘Chimeric Antigen Receptor’ AND ‘Diffuse Large B-Cell Lymphoma’ AND ‘Cytokine Release Syndrome’. In the PubMed database the search was refined by document type as ‘Clinical Trial’ and in the Scopus and Web of Science databases refined as ‘Article’.

### Inclusion and exclusion criteria

Clinical trials of patients with DLBCL who received CAR-T cell therapy were included with no restrictions on age, gender, ethnicity, or presence of comorbidities.

Studies in which the principal diagnosis was not DLBCL were excluded. Patients who received other concomitant therapies during the clinical trial (except for lymphodepleting chemotherapy and bridging therapy) were excluded. Studies that did not report the incidence of CRS were excluded as were duplicate studies.

### Data extraction

The database search was performed independently by two authors who subsequently screened the title and abstract of the articles. Studies that met the inclusion criteria were included for full-text review. After reading the full text, the studies were assessed for eligibility; in cases where the two evaluators did not agree, a third evaluator was used to arrive at a conclusion.

### Data overview

Data from the included studies were extracted in a predetermined format and individually presented in table format. The following data were extracted from each study: study characteristics (lead author, year of publication, study location, study period, and sample size), patient characteristics (mean age, number of previous chemotherapy lines), treatment characteristics (target chimeric antigen receptor antigen, lymphodepletion and bridging therapy regimens, CAR-T cell product and dose, overall response and relapse-free survival) and CRS characteristics (occurrence, grade, deaths, time of symptom onset, duration, management). Data were not meta-analyzed.

## Results

**Figure 1:**
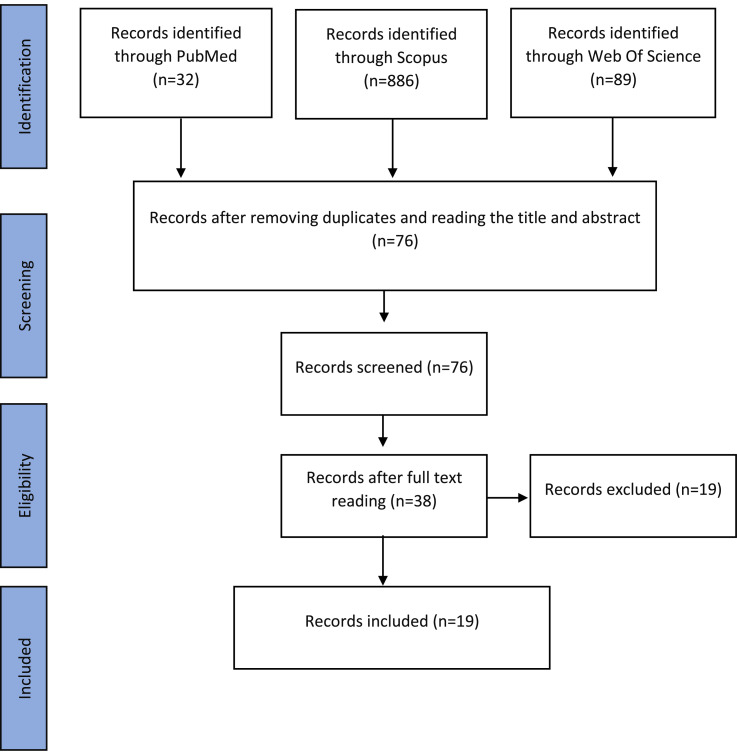
Flowchart of article selection.

Figure 1 shows that the systematic search in the three databases resulted in the identification of 1007 articles. After excluding duplicates and after screening by title and abstract, 76 articles were read in full. Consequently, 19 studies that fulfilled the eligibility criteria were included in this review.

The articles included in this review were published between 2017 and 2022, with China (*n* = 7) producing the most studies, followed by United States of America (USA) (*n* = 6) and Japan (*n* = 4). A total of 1193 patients enrolled in 19 clinical trials received CAR-T cell therapy. The sample sizes ranged from 7 to 256 patients across studies.

The ages of the 1193 patients ranged from 17 to 86 years with a mean age of 58 years. Interestingly, the PILOT study by Sehgal et al.^26^ from the USA that included 61 participants, recruited only over 70-year-old patients. Most studies (*n* = 11) included patients who had received at least two lines of prior therapy, another five studies required at least one line of prior therapy and three studies tested CAR-T cell therapy as second-line (Table 1).

**Table 1:** Characteristics of the studies and enrolled patients.

Author and year	Study site	Period	Study type	n	Average age in years (range)	Lines of previous therapies
Schuster et al. (2019)^11^	27 locations in 10 countries in North America, Europe, Australia and Asia	Jul 2015 - Dec 2017	Multicenter Phase II - JULIET	111	56 (22–76)	≥1
Neelapu et al. (2017)^12^	22 study centers (21 in the USA and 1 in Israel)	Nov 2015 - Sept 2016	Phase II Multicenter - ZUMA-1	101	58 (23–76)	≥2
Abramson et al. (2020)^13^	14 cancer centers in the USA	Jan 2016 - Jul 2019	Multicenter phase I - TRANSCEND	256	63 (54–70)	≥2
Neelapu et al. (2022)^14^	7 medical centers in the USA, Australia and France	Fev 2019 - Oct 2020	Phase II Multicenter - ZUMA 12	40	61 (23–86)	≥2
Kato et al. (2021)^15^	Japan	Jul 2019 - Oct 2019	Phase II multicenter	16	58 (44–70)	≥2
Ying et al. (2020)^16^	China	Nov 2017 - Dec 2019	Multicenter, open and single-arm	59	56 (18–75)	≥2
Locke et al. (2017)^17^	4 centers in the USA	Apr 2015 - Aug 2016	Phase I Multicenter - ZUMA-1	7	52 (29–69)	≥2
Makita et al. (2022)^18^	Japan	Data cutting Jun 2020	Multicenter phase I - TRANSCEND	10	57 (47–73)	≥1
Huang et al. (2020)^19^	China	Unreported	Single Center	11	49 (29–69)	≥2
Goto et al. (2020)^20^	Japan	Data hack May 2018	Multicenter -Phase II single arm - JULIET	9	61 (41–73)	≥2
Zhang et al. (2021a)^21^	China	Set 2018 - Feb 2020	Single arm and single center	31	73 (65–86)	≥1
Locke et al. (2022a)^22^	77 locations worldwide	Jan 2018 - Oct 2019	International Phase III - ZUMA-7	170	58 (21–80)	1
Cheng et al. (2022)^23^	China	Nov 2017 - Dec 2021	Single Phase I centre	15	48 (30–66)	≥2
Kedmi et al. (2022)^24^	Israel	Nov 2017 - Dec 2020	Step Ib/II	72	49 (20–73)	≥2
Kamdar et al. (2022)^25^	47 cancer medical centers in the USA, Europe and Japan	Oct 2018 - Dec 2020	Pivotal, global, open phase III	83	60 (53–67)	1
Sehgal et al. (2022)^26^	18 clinical centers in the USA	Jul 2018 - Sept 2021	Phase II open - PILOT	61	74 (70–78)	1
Zhang et al. (2021b)^27^	China	May 2017 - Jan 2020	Open phase I/II single arm	87	50 (17–68)	≥2
Qu et al. (2022)^28^	China	Jun 2017 - Apr 2022	Single phase II Center	33	55 (31–72)	≥1
Sang et al. (2020)^29^	China	Mar 2017 - Oct 2018	Single phase II Center	21	55 (23–72)	≥1

The characteristics of the CAR-T cell treatment are listed in Table 2. Regarding the CAR-T cell product used, seven studies (37%) did not use commercially described products, five studies (26%) used axicabtagene ciloleucel, four (21%) used lisocabtagene maraleucel, two (11%) used tisagenlecleucel and one (5%) used relmacabtagene autoleucel. The target chimeric antigen receptor antigen in 16 studies (84%) was CD19, in another two studies (11%) it was CD19 plus CD22 and one study (5%) targeted the CD20 antigen.

**Table 2:** Characteristics of the CAR-T cell intervention.

Reference	CAR-T cell therapy Trade Name	Target CAR antigen	Lymphodepletion therapy -%	Lymphodepletion regime	Patients who received bridge therapy%	Bridge therapy regimen	CAR-T dose applied (cells)	Full response%	Progression-free survival in months
Schuster et al. (2019)^11^	Tisagenlecleucel	CD19	93	Fludarabine + cyclophosphamide or bendamustine	92	Systemic therapy	3.0 × 10^8^/kg	33	not reached
Neelapu et al. (2017)^12^	Axicabtagene ciloleucel	CD19	100	Fludarabine + cyclophosphamide	0	not allowed	2.0 × 10^6^/kg	54	5.8
Abramson et al. (2020)^13^	Lisocabtagene maraleucel	CD19	100	Fludarabine + cyclophosphamide	59	Systemic therapy and/or radiotherapy	91 × 10^6^	53	6.8
Neelapu et al. (2022)^14^	Axicabtagene ciloleucel	CD19	100	Fludarabine + cyclophosphamide	17	Systemic therapy	2.0 × 10^6^/kg	78	not reached
Kato et al. (2021)^15^	Axicabtagene ciloleucel	CD19	100	Fludarabine + cyclophosphamide	0	not allowed	2.0 × 10^6^/kg	27	6.5
Ying et al. (2020)^16^	Relmacabtagene autoleucel	CD19	100	Fludarabine + cyclophosphamide	44	Not disclosed	100 × 10^6^ (low dose) or 150 × 10^6^ (high dose)	52	7
Locke et al. (2017)^17^	Axicabtagene ciloleucel	CD19	100	Fludarabine + cyclophosphamide	0	Not disclosed	2.0 × 10^6^/kg	57	Unreported
Makita et al. (2022)^18^	Lisocabtagene maraleucel	CD19	100	Fludarabine + cyclophosphamide	100	Not disclosed	100 × 10^6^	50	6.3
Huang et al. (2020)^19^	a non=commercial product	CD19	100	Fludarabine + cyclophosphamide	0	Not disclosed	1.8 to 3.0 × 10^6^/kg	64	not reached
Goto et al. (2020)^20^	Tisagenlecleucel	CD19	100	Fludarabine + cyclophosphamide or bendamustine	67	Systemic therapy	2.0 × 10^8^/kg	56	Not disclosed
Zhang et al. (2021a)^21^	a non=commercial product	CD19	100	Fludarabine + cyclophosphamide	0	Not disclosed	2.0 × 10^6^/kg	52	11.4
Locke et al. (2022a)^22^	Axicabtagene ciloleucel	CD19	100	Fludarabine + cyclophosphamide	36	Glucocorticoids	2.0 × 10^6^/kg	65	14.7
Cheng et al. (2022)^23^	a non-commercial product	CD20	87	Fludarabine + cyclophosphamide	0	not allowed	1.0 × 10^7^/kg	60	not reached
Kedmi et al. (2022)^24^	a non=commercial product	CD19	100	Fludarabine + cyclophosphamide	8	Systemic therapy	1.0 × 10^6^/kg	37	3.7
Kamdar et al. (2022)^25^	Lisocabtagene maraleucel	CD19	100	Fludarabine + Cyclophosphamide	63	Immunochemotherapy	100 × 10^6^	66	10.1
Sehgal et al. (2022)^26^	Lisocabtagene maraleucel	CD19	100	Fludarabine + Cyclophosphamide	52	Not disclosed	100 × 10^6^	54	22.6
Zhang et al. (2021b)^27^	a non=commercial product	CD19	100	Fludarabine + Cyclophosphamide	Unreported	Not disclosed	0.5 × 10^6^ to 8 × 10^6^/kg	70	27.6
Qu et al. (2022)^28^	a non=commercial product	CD19/ CD22	100	Decitabine + Fludarabine + Cyclophosphamide	Unreported	Not disclosed	1 × 10^7^/kg	39	10.2
Sang et al. (2020)^29^	a non=commercial product	CD19/ CD22	100	Fludarabine + Cyclophosphamide or Ifosfamide	0	Not disclosed	1 × 10^6^/kg	40	5

CAR: chimeric antigen receptors.

Lymphodepleting therapy was administered in 100% of patients in 17 studies (89%), one study (5%) performed lymphodepletion in 93% of patients, and the other study (5%) in 87% of patients. The regimen employed for lymphodepletion was fludarabine plus cyclophosphamide in 15 studies (79%), fludarabine plus cyclophosphamide or bendamustine in two studies (11%), fludarabine plus cyclophosphamide and decitabine in one study (5%), and fludarabine plus cyclophosphamide and ifosfamide in one study (5%). The doses and administration regimens were quite variable as can be seen in more detail in Table 3.

**Table 3:** Lymphodepletion regimen.

Reference	Drug	Dose	Scheme	CAR-T infusion
Neelapu et al. (2017)^12^ Neelapu et al. (2022)^14^ Kato et al. (2021)^15^ Locke et al. (2017)^17^ Locke et al. (2022a)^22^ Kamdar et al. (2022)^25^	Fludarabine plus Cyclophosphamide	(30 mg/m²/day) + (500 mg/m²/day)	For 3 consecutive days	2 days after
Schuster et al. (2019)^11^	Fludarabine plus cyclophosphamide or bendamustine	(25 mg/m²/day) + (500 mg/m²/day) or (90 mg/m²/day)	For 3 consecutive days For 2 consecutive days	2 days after
Goto et al. (2020)^20^	Fludarabine plus cyclophosphamide or bendamustine	(25 mg/m²/day) + (250 mg/m²/day) or (90 mg/m²/day)	For 3 consecutive days For 2 consecutive days	2 days after
Ying et al. (2020)^16^	Fludarabine plus cyclophosphamide	(25 mg/m²/day) + (250 mg/m²/day)	For 3 consecutive days	2–7 days after
Huang et al. (2020)^19^	Fludarabine plus cyclophosphamide	(25 mg/m²/day) + (900 mg/m²/day)	Fludarabine D1 to D3 Cyclophosphamide D3 and D4,	2 days after
Cheng et al. (2022)^23^	Fludarabine plus cyclophosphamide	(25 mg/m²/day) + (500 mg/m²/day)	For 3 consecutive days	2–7 days after
Kedmi et al. (2022)^24^	Fludarabine plus cyclophosphamide	(25 mg/m²/day) + (900 mg/m²/day)	Fludarabine on days 4 to 2 Cyclophosphamide on day 2	2 days after
Abramson et al. (2020)^13^	Fludarabine plus cyclophosphamide	(30 mg/m²/day) + (300 mg/m²/day)	For 3 consecutive days	2 days after
Makita et al. (2022)^18^ Sehgal et al. (2022)^26^	Fludarabine plus cyclophosphamide	(30 mg/m²/day) + (300 mg/m²/day)	For 3 consecutive days	2–7 days after
Zhang et al. (2021b)^27^	Fludarabine plus cyclophosphamide	(20–30 mg/m²/day) + (20–30 mg/kg over 3 days)	For 3 consecutive days	2 days after
Zhang et al. (2021a)^21^	Fludarabine plus cyclophosphamide	(30 mg/m²/day) + (300 mg/m²/day)	For 3 consecutive days	2–4 days after
Qu et al. (2022)^28^	Decitabine plus cyclophosphamide plus fludarabine	(100 mg/m²/day) + (300 mg/m²/day) + (30 mg/m²/day)	For 3 consecutive days	2 days after
Sang et al. (2020)^29^	Fludarabine plus cyclophosphamide ifosfamide	(30 mg/m²/day) + (750 mg/m²/day) + (2 g/day)	Fludarabine for 3 days Cyclophosphamide for one day Ifosfamide for 3 days	2 days after

Bridging therapy was used in ten studies (53%), the highest frequencies of the use of this therapeutic modality were reported in the articles by Makita et al.^18^ (where all patients received bridging therapy), by Schuster et al.^11^ (92% of patients) and by Kamdar et al.^25^ (63%). The most commonly employed bridging regimen was systemic therapy with glucocorticoids or chemotherapy described in seven studies (37%); one study (5%) employed radiation therapy in addition to systemic therapy, in three studies (16%) bridging therapy was not allowed, two (11%) had no need to use bridging therapy, and seven studies (37%) did not report the regimen that was or would be employed.

The doses of CAR-T applied varied greatly among the studies, 14 studies applied doses based on the weight (kg) of the patient; the doses in these studies ranged from 0.5 × 10^6^ to 3.0 × 10^8^ CAR-T cells/kg. The other five studies applied a fixed average dose that ranged from 91.0 × 10^6^ to 150.0 × 10^6^ CAR-T cells. All patients received a single infusion of CAR-T cells, except in one study where the dose was split over three days in a staggered manner (10%, 30%, and 60% of the total dose).

The outcomes of the studies had a median complete response of 54%; the study with the highest complete response was by Neelapu et al. (the ZUMA-12 study)^14^ with 78% of patients achieving a complete response. The worst outcome was seen in the report by Kato et al.^15^ where only 27% of patients achieved a complete response.

Progression-free survival ranged from 5 to 27.6 months, with a median of seven months. In four (21%) studies progression-free survival was not achieved due to the short follow-up period, and two studies (11%) did not report information on progression-free survival.

The overall results of the research related to CRS are presented in Table 4. In this review, the CRS did not receive a standard classification but rather the classification was determined by original articles. It is noteworthy that significant discrepancies may exist between various classification systems currently employed for toxicity related to CAR-T cell therapy. This disparity can have implications on the diagnosis and treatment of CRS.

**Table 4:** Characteristics of cytokine release syndrome.

Reference	n	Occurrence of any degree CRS (%)	Grade 3 CRS (%)	Grade 4 CRS (%)	Deaths due to CRS (%)	Average time of onset of symptoms in days (range)	Average duration in days (range)	Management
Schuster et al. (2019)^11^	111	64 (58)	15 (14)	9 (8)	0	3 (2–9)	7 (2–30)	14% received tocilizumab and 10% received tocilizumab and glucocorticoids
Neelapu et al. (2017)^12^	101	94 (93)	9 (9)	4 (4)	2 (2%)	2 (1 −12)	8 (Not disclosed)	43% received tocilizumab and 27% received glucocorticoids
Abramson et al. (2020)^13^	256	113 (42)	4 (1)	2 (1)	0	5 (1–14)	5 (1–17)	10% received only tocilizumab, 8% Tocilizumab and corticosteroids and 2% only glucocorticoids
Neelapu et al. (2022)^14^	40	40 (100)	3 (8)	Not disclosed	0	4 (1 −10)	6 (Not disclosed)	63% received tocilizumab and 35% received glucocorticoids
Kato et al. (2021)^15^	16	13 (81)	0	1 (8)	0	2 (1–11)	16.5 (Not disclosed)	68.8% received tocilizumab and 56.3% received glucocorticoids
Ying et al. (2020)^16^	59	28 (47)	2 (3)	1 (2)	0	4.5 (1–10)	7 (1–118)	27% received tocilizumab and 10% received glucocorticoids
Locke et al. (2017)^17^	7	6 (86)	0	1 (14)	0	1 (0–3)	7 (3–17)	86% received tocilizumab, 57% received glucocorticoids
Makita et al. (2022)^18^	10	5 (50)	0	0	0	3 (2–9)	4 (1–5)	20% received tocilizumab, 10% received glucocorticoids
Huang et al. (2020)^19^	11	10 (91)	0	0	0	Not disclosed	Not disclosed	27% received glucocorticoids
Goto et al. (2020)^20^	9	6 (67)	1 (11)	1 (11)	0	4 (1 −8)	7.5 (4–11)	33% received tocilizumab
Zhang et al. (2021a)^21^	31	16 (52)	4 (13)	0	Not disclosed	4.6 (3–7)	Not disclosed	Not disclosed
Locke et al. (2022a)^22^	170	157 (92)	11 (6)	0	0	3 (1–10)	7 (2–43)	65% received tocilizumab, 24% received glucocorticoids
Cheng et al. (2022)^23^	15	15 (100)	5 (33)	0	0	0.63 (0.25–1.3)	6 (1–15)	47% received tocilizumab, 40% received glucocorticoids
Kedmi et al. (2022)^24^	72	62 (85)	5(7)	2 (3)	0	4 (2–5)	5 (4–9)	7% received tocilizumab, 6% received tocilizumab and glucocorticoids
Kamdar et al. (2022)^25^	83	45 (49)	1 (1)	0	0	5 (3–8)	4 (2–5)	10% received tocilizumab, 13% received tocilizumab and glucocorticoids
Sehgal et al. (2022)^26^	61	23 (38)	1 (2)	0	0	4 (3–7)	4 (2–5)	10% received tocilizumab, 16% received tocilizumab and glucocorticoids
Zhang et al. (2021b)^27^	87	61 (70)	8 (9)	1 (1)	Not disclosed	1 (1–9)	6 (1–11)	25% received monotherapy or combination therapy with tocilizumab, infliximab, etanercept and glucocorticoids
Qu et al. (2022)^28^	33	25 (76)	5 (21)	0	Not disclosed	Not disclosed	Not disclosed	12% received tocilizumab and 16% received glucocorticoids
Sang et al. (2020)^29^	21	21 (100)	6 (28)	Not disclosed	0	2 (0–5)	5 (2–14)	19% received glucocorticoids

Of the 1193 patients included in this review, 804 (67%) developed some degree of CRS. The mean frequency of occurrence of any degree of CRS was 72%. In three studies, all patients developed CRS. The study with the lowest occurrence of CRS was Abramson et al.^13^ in which 113 patients (42%) had some degree of CRS.

Grade 3 CRS was reported in 80 (10%) patients of the 804 patients who evolved with some grade of CRS. The studies with the highest frequencies were Cheng et al.^23^, Sang et al.^29^ and Qu et al.^28^ (Grade 3 CRS in 33%, 28% and 21% of patients, respectively). No occurrence of Grade 3 CRS was reported by four studies.

Grade 4 CRS was reported in 22 (3%) patients of the 804 patients who developed some degree of CRS. Two studies did not report information about the occurrence of Grade 4 CRS. In eight studies, there were no cases of Grade 4 CRS. The highest frequencies of Grade 4 CRS were observed in the studies by Locke et al.^17^ (14%), Goto et al.^20^ (11%), Kato et al.^15^ (8%) and Schuster et al. (8%).^11^

Only one study recorded death as a result of CRS, with 2% of CRS-associated deaths, that is, 0.25% of the 804 patients who developed some degree of CRS.

The mean time of onset of CRS symptoms ranged from 0.63 to 5 days, with a median of three days. The greatest variation was found in the study by Abramson et al.^13^ who recorded a range of 1–14 days. Two studies did not report information on the time of symptom onset. The mean duration of CRS ranged from 4 to 16.5 days, with a median of six days. The greatest variation was recorded by Ying et al.^16^, where the time to solve CRS ranged from 1 to 118 days. Three studies reported no information on the duration of CRS.

The regimen employed for the management of CRS was based on the use of tocilizumab and/or glucocorticoids in 14 studies, two studies used glucocorticoids only, one study used tocilizumab only, and one other study reported using monotherapy or combination therapy with tocilizumab, infliximab, etanercept, and glucocorticoids. One study did not report the therapeutic regimen employed.

## Discussion

Our analysis shows that most patients develop CRS (mean frequency 72%), but only 10% and 3% evolve with Grade 3 or 4 CRS, respectively. Hernani et al.^30^ presented similar data when they reported that CRS affects from 42 to 93% of CD19 with severe (Grade ≥ 3) CRS in 2–22%.

Analyzing the data obtained in this review there seems to be no correlation between the average age of patients and the development of CRS. An even more curious finding was that the study by Sehgal et al.^26^, which only enrolled over 70-year-old patients, reported the lowest frequency of CRS of all the included studies. These findings differ from the results of Locke et al.^31^ who found that older patients with DLBCL had a higher risk of CRS or neurotoxicity syndrome when treated with axicabtagene ciloleucel.

The development of CRS and the type of commercial CAR-T product applied seem to be related. While studies using lisocabtagene maraleucel had an average occurrence of CRS of 45%, those using tisagenlecleucel had an average of 63%, and those using axicabtagene ciloleucel had an average of 90%. Supporting these data, a study conducted by the Spanish Lymphoma and Bone Marrow Transplant Group of patients with refractory DLBCL demonstrated that axicabtagene ciloleucel may be superior in efficacy to tisagenlecleucel, yet more toxic.^32^

The combination of fludarabine and cyclophosphamide was the most commonly used conditioning therapy. Studies point out that conditioning chemotherapy is essential for the efficacy of adoptive T-cell therapy. The addition to fludarabine, cyclophosphamide has been associated with better CAR-T cell expansion and persistence compared to other regimens, and the combination of these agents represents the preferred conditioning regimen in patients with relapsed or refractory DLBCL.^33^

A Phase II clinical trial demonstrated that radiotherapy is an optimal volume reducing regimen for the management of patients with DLBCL prior to CAR-T cell therapy and a promising alternative salvage therapy for patients who relapse after CAR-T cell therapy.^34^ In this review, none of the included studies used radiotherapy as conditioning.

Bridge therapy has varied widely, from standard chemotherapy to targeted therapies (immunomodulators, tyrosine kinase inhibitors), to radiation and finally corticosteroids. Studies show that bridging therapy is used in about half of individuals.^33^ Cook et al.^35^ stress that most patients with lymphoma require bridging therapy while waiting for CAR-T cells to be produced due to the long time between the initial evaluation of the patient to the infusion of the cells.

Bridging therapy remains very heterogeneous, and no preferable strategy has been identified to date.^36^ In this review, we identified one study that used, in addition to systemic therapy, radiation as bridging therapy. Recent research demonstrates that radiation therapy is a safe and effective method for patients with DLBCL, not only because it reduces myelosuppression compared to bridging chemotherapy, but also it potentially improves the tumor microenvironment.^37^

The conditioning regimen may affect the development of CRS. Wang et al.^38^ reported low toxicity in treating B-cell non-Hodgkin lymphoma patients with central memory-derived CAR-T cells after autologous transplantation, suggesting that certain T-cell subsets and the choice of conditioning therapy prior to CAR-T infusion contribute to the severity of CRS.

The data set obtained in this review shows a safety profile regarding CRS that is similar to the published literature. As initially discussed, we observed in this review a high rate of CRS, but the portion of patients who evolve to a more severe degree is relatively small. Still in this regard, the number of deaths from CRS was also low. However, it is still necessary to consider that the causes of CRS are very heterogeneous and different classification systems increase the differences in CRS rates between studies.^39^

The use of tocilizumab appears to be well established as a treatment for CRS. Studies show that tocilizumab, an interleukin-6 blocking therapy, resulted in rapid recovery from CRS in patients with aggressive non-Hodgkin lymphoma treated with CAR-T. Some authors also advocate the early use of tocilizumab as a strategy to reduce the risk of severe CRS further. Steroid therapy may also be effective in attenuating CRS and is currently reserved as second line therapy after tocilizumab failure.^39^^,^^40^

Cook et al.^35^ point out that future studies should seek to better elucidate the mechanisms of these toxicities and develop prognostic models to identify subsets of patients who will benefit from prophylactic strategies to prevent immune effector cell-associated toxicities. A deeper understanding of CRS will allow the development of new approaches to reduce toxicities and improve the outcomes of CAR-T cell therapy.^39^

## Conclusion

The recent approval of CAR-T cell therapy has marked a new era in cancer treatment. This therapy has been shown to cure some patients and extend survival for many others whose treatment options would otherwise be limited. However, this success comes with challenges as is the case with CRS. The results obtained in this review demonstrate high rates of CRS in DLBCL patients treated with CAR-T cell therapy nevertheless these adverse events are manageable, supporting the conclusion that this therapy is safe in DLBCL patients.

## Funding

This research did not receive any specific grant from funding agencies in the public, commercial, or not-for-profit sectors.

## Conflicts of interest

Authors declare no conflicts of interest.
